# Efficacy of nanoparticle albumin-bound paclitaxel regimens for relapsed small cell lung cancer: A retrospective analysis

**DOI:** 10.1097/MD.0000000000007884

**Published:** 2017-09-01

**Authors:** Yujiro Naito, Akihiro Tamiya, Motohiro Tamiya, Yohei Kimura, Masanari Hamaguchi, Nobuhiko Saijo, Masaki Kanazu, Sayoko Tokura, Takayuki Shiroyama, Naoko Morisita, Naoki Omachi, Hidekazu Suzuki, Norio Okamoto, Kyoichi Okishio, Tomonori Hirashima, Shinji Atagi

**Affiliations:** a Department of Internal Medicine, National Hospital Organization Kinki-chuo Chest Medical Center; b Department of Respiratory Medicine and Clinical Immunology, Graduate School of Medicine, Osaka University, Suita City; c Department of Thoracic Oncology, Osaka Prefectural Hospital Organization Osaka Habikino Medical Center, Habikino City; d Department of Respiratory Internal Medicine, Osaka International Cancer Institute; e Department of Thoracic Oncology, National Hospital Organization Toneyama National Hospital, Toyonaka City; f Department of Thoracic Oncology, National Hospital Organization Kinki-chuo Chest Medical Center, Sakai City, Osaka, Japan.

**Keywords:** carboplatin, chemotherapy, nab-paclitaxel, relapse, small cell lung cancer

## Abstract

Although small cell lung cancer (SCLC) is initially sensitive to chemotherapy, it recurs in most cases. Standard regimens for salvage chemotherapy have not been established, and the prognosis of relapsed SCLC remains poor. In the present study, we investigated the clinical efficacy and safety of nanoparticle albumin-bound paclitaxel (nab-paclitaxel) regimens for the treatment of relapsed SCLC.

In this retrospective multicenter analysis, 14 patients (3 women and 11 men; median age 71 years) with relapsed SCLC received nab-paclitaxel alone or in combination with carboplatin between February 2013 and July 2014. The safety and efficacy of the regimens were evaluated.

The response rates, disease control rates, and median overall survival for the total patient population were 36%, 64%, and 7.8 months, respectively. Response rates, disease control rates, and the median overall survival were 11%, 44%, and 4 months, respectively, in the monotherapy group; and 80%, 100%, and 10.6 months, respectively, in the combination therapy group. The most common adverse events were hematological toxicities such as neutropenia and anemia. Severe neutropenia appeared in some patients, although it was resolved by treatment in all. The most common nonhematological toxicity was anorexia (64%), followed by neurotoxicity and constipation. All nonhematological toxicities were mild and manageable.

Our results suggest that chemotherapy with nab-paclitaxel regimens for relapsed SCLC exhibits moderate clinical efficacy and is well-tolerated. Further clinical trials in relapsed SCLC patients are warranted.

## 1 Introduction

Small cell lung cancer (SCLC) is the most rapidly growing subtype of lung cancer, and patient prognosis is extremely poor.^[1]^ Although SCLC is initially sensitive to chemotherapy, it recurs in most cases.

Second-line chemotherapy has been reported to alleviate symptoms and prolong the survival of patients with relapsed SCLC.^[2]^ To date, various agents have been reported as effective for the management of relapsed SCLC, including topotecan, amrubicin, etoposide, irrinotecan, and gemcitabine, among others.^[2–7]^ However, standard regimens for salvage chemotherapy have not been established, and the prognosis of relapsed SCLC remains poor. Therefore, effective treatments to improve the prognosis of patients with relapsed SCLC are desirable.

The efficacy of paclitaxel regimens (paclitaxel alone or in combination with carboplatin [CBDCA]) for both untreated and relapsed SCLC has been reported in some studies.^[8–11]^ However, because of the lack of adequate data, paclitaxel regimens are not considered the mainstay of chemotherapy for SCLC and are generally administered when other agents prove ineffective.

Nanoparticle albumin-bound paclitaxel (nab-paclitaxel [Abraxane]) is a formulation comprising paclitaxel bound to human serum albumin. Preclinical models suggest that nab-paclitaxel may reach the tumor microenvironment more efficiently than paclitaxel, and may be preferentially taken up by cancer cells.^[12]^ Compared with paclitaxel, nab-paclitaxel has advantages such as effective migration to the tumor, fewer allergies, and lesser neurotoxicity. It has shown good activity in various advanced solid tumors, including breast cancer, melanoma, pancreatic cancer, and nonsmall cell lung cancer (NSCLC).^[12–14]^ Furthermore, in clinical studies of NSCLC, nab-paclitaxel increased the overall response rate (RR) and produced less neuropathy compared with paclitaxel.^[15]^

On the basis of these results, we hypothesized that nab-paclitaxel is effective and well-tolerated in patients with relapsed SCLC. However, to our knowledge, there are only 2 reports concerning SCLC and nab-paclitaxel: one is about monotherapy for relapsed SCLC and the other is about combination with CBDCA for untreated SCLC.^[16,17]^ Since the data obtained in these 2 papers are insufficient to determine the role of nab-paclitaxel for SCLC, it is necessary to evaluate further. Therefore, we conducted this retrospective study to evaluate the efficacy and safety of nab-paclitaxel alone and in combination with CBDCA for relapsed SCLC.

## 2 Materials and methods

This study was conducted in accordance with the principles of the Declaration of Helsinki and was approved by the institutional review boards for human experimentation at Osaka Prefectural Medical Center for Respiratory and Allergic Diseases and Kinki-chuo Chest Medical Center.

### 2.1 Patient selection and data collection

We retrospectively surveyed the databases at the 2 hospitals and enrolled 14 patients with histologically and cytologically confirmed SCLC who were treated with nab-paclitaxel regimens between February 2013 and November 2014 because of disease progression after previous standard chemotherapy. All patients exhibited an Eastern Cooperative Oncology Group Performance Status (ECOG PS) of 1 to 2, and lesions were measurable with Response Evaluation Criteria in Solid Tumors (RECIST) version 1.1.^[18]^

Data for patient characteristics, treatment efficacy, and adverse events were retrospectively collected from the medical records of all patients. The cut-off date for this analysis was August 7, 2015.

### 2.2 Treatment regimens

Patients were treated with nab-paclitaxel alone or in combination with CBDCA. The monotherapy group received 100 mg/m^2^ nab-paclitaxel administered weekly for 3 weeks in a 4-week cycle or weekly for 2 weeks in a 3 to 4-week cycle. The combination group received CBDCA (area under the curve [AUC] 6) plus 100 mg/m^2^ nab-paclitaxel weekly for 3 weeks in a 4-week cycle or for 2 weeks in a 3 to 4-week cycle. The chemotherapy regimen and administration dose were determined on the basis of the patient condition by the physician in charge. Each treatment cycle was repeated 4 to 6 times unless there was evidence of disease progression or unacceptable toxicity, or the patient/physician decided to discontinue treatment. Subsequent doses were modified by the physician in charge on the basis of hematological and nonhematological toxicities.

The organ function of all patients was adequate before treatment initiation, and they had provided written informed consent to undergo treatment. Peripheral blood and biochemistry examinations were repeated at least once per cycle.

### 2.3 Evaluations

All patients were evaluated to determine the disease stage before treatment initiation and at the time of determination of disease progression or relapse through complete medical histories and physical examinations, chest radiography, chest and abdominal computed tomography (CT), and other staging procedures such as head magnetic resonance imaging (MRI).

Limited disease (LD) was defined as that confined to 1 hemithorax and including the bilateral mediastinal and supraclavicular nodes. Disease extending beyond these boundaries was defined as extensive disease (ED).

Primary refractory disease (refractory relapse) was defined as relapse during or less than 90 days after completion of first-line chemotherapy regimen, whereas sensitive disease (sensitive relapse) was defined as relapse more than 90 days after completion of first-line chemotherapy.

The tumor response was classified in accordance with RECIST version 1.1.^[18]^ Adverse events were recorded and graded using the grading system of the Common Terminology Criteria for Adverse Events version 4.0.^[19]^

### 2.4 Statistical analysis

Progression-free survival (PFS) was defined as the time from the first administration of the nab-paclitaxel regimen to the date of confirmation of disease progression or death. Overall survival (OS) was defined as the time from the first administration of the nab-paclitaxel regimen until death. Data for patients with unavailable information regarding death or disease progression were censored at the date of the last assessment.

The median probabilities for PFS and OS were estimated by the Kaplan–Meier method. The time-to-event outcomes were compared using log-rank tests. All analyses were performed using JMP 11 statistical software (SAS Institute, Cary, NC).

## 3 Results

In total, 14 patients (3 women and 11 men; median age 71 years; range 58–83 years) with relapsed SCLC received weekly nab-paclitaxel or CBDCA plus nab-paclitaxel chemotherapy between February 2013 and November 2014 at our hospitals. All patients were assessable for efficacy and safety. The patient characteristics are shown in Table 1. Eight patients exhibited comorbid pulmonary disease, including interstitial lung disease (ILD) in 5, chronic obstructive pulmonary disease (COPD) in 2, and both ILD and COPD in 1. Three patients exhibited LD and 11 exhibited ED at the start of treatment. Twelve and 2 patients exhibited refractory and sensitive relapse, respectively.

**Table 1:**
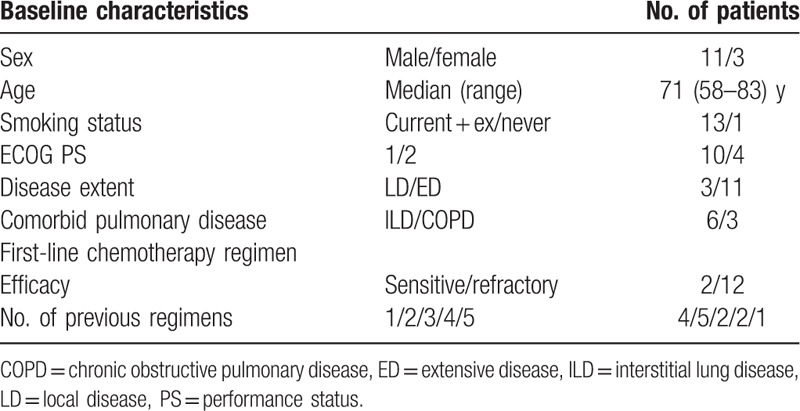
Characteristics of patients with relapsed small cell lung cancer included in the present study.

Table 2 shows the previous chemotherapy regimens received by the 14 patients. All patients had received platinum doublet regimens. Four received nab-paclitaxel regimens as second-line chemotherapy, 5 as third-line chemotherapy, and 5 as fourth-line or later chemotherapy. Nine patients were treated with nab-paclitaxel alone (monotherapy group) and 5 were treated with CBDCA plus nab-paclitaxel (combination therapy group). One patient in the monotherapy group and all patients in the combination therapy group exhibited ILD. The details of the chemotherapy regimens are shown in Table 3. Nine patients required dose skipping or dose reduction. The median number of administration cycles was 3.

**Table 2:**
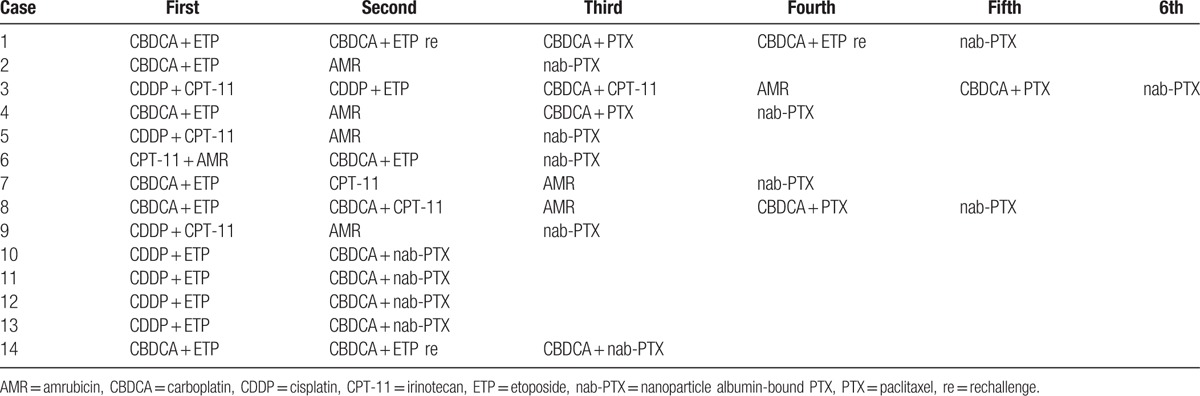
Previous chemotherapy regimens for the patients with relapsed small cell lung cancer included in the present study.

**Table 3:**
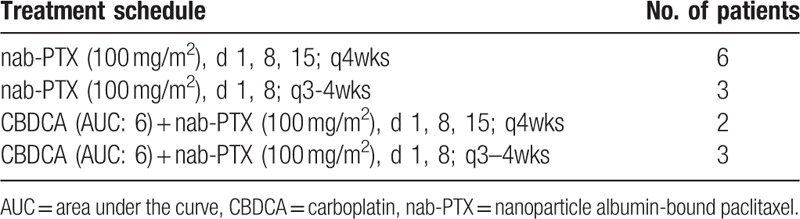
Treatment schedule for the patients with relapsed small cell lung cancer included in the present study.

**Figure 1:**
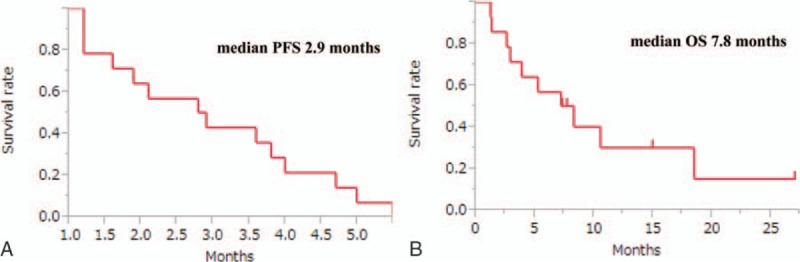
(A) Kaplan–Meier curves for progression-free survival (PFS); and (B) overall survival (OS) rates for the overall patient population with relapsed small cell lung cancer.

**Figure 2:**
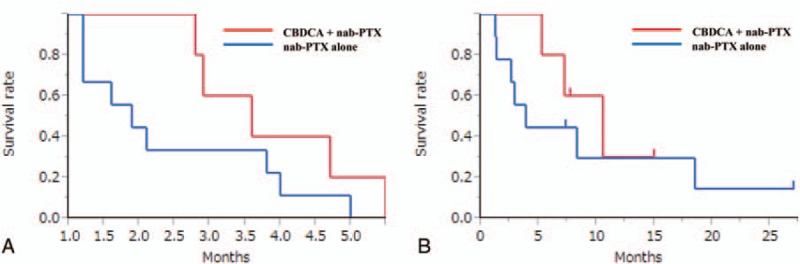
(A) Kaplan–Meier curves for progression-free survival and (B) overall survival (OS) rates for patients who received nab-paclitaxel (nab-PTX) alone and those who received carboplatin plus nab-PTX (CBDCA + nab-PTX) for relapsed small cell lung cancer.

Five patients achieved partial response and 4 exhibited stable disease. RR, the disease control rate (DCR), the median PFS, and the median OS were 36%, 64%, 2.9 months, and 7.8 months for the total patients population (Fig. 1). Figure 2 and Table 4 show the treatment efficacy in the monotherapy and combination therapy groups. RR, DCR, and the median OS were 11%, 44%, and 4 months, respectively, in the monotherapy group; and 80%, 100%, and 10.6 months, respectively, in the combination therapy group.

**Table 4:**
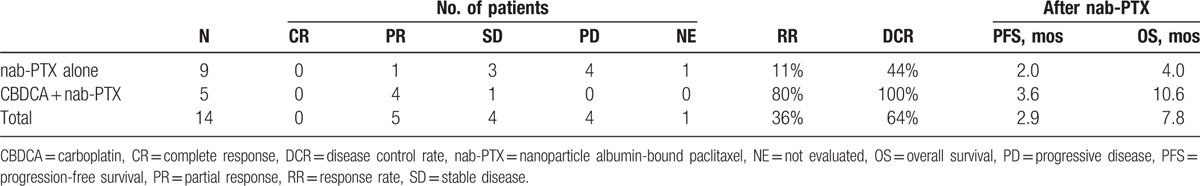
Efficacy of chemotherapy with nanoparticle albumin-bound paclitaxel alone and nanoparticle albumin-bound paclitaxel plus carboplatin in patients with relapsed small cell lung cancer included in the present study.

The most common adverse events were hematological toxicities such as neutropenia and anemia (Table 5). Severe neutropenia (grade 3 or 4) appeared in some patients, although it was resolved by treatment in all. The most common nonhematological toxicity was anorexia (64%), followed by neurotoxicity and constipation. Pneumonitis and acute exacerbation of ILD did not occur. All nonhematological toxicities were mild and manageable.

**Table 5:**
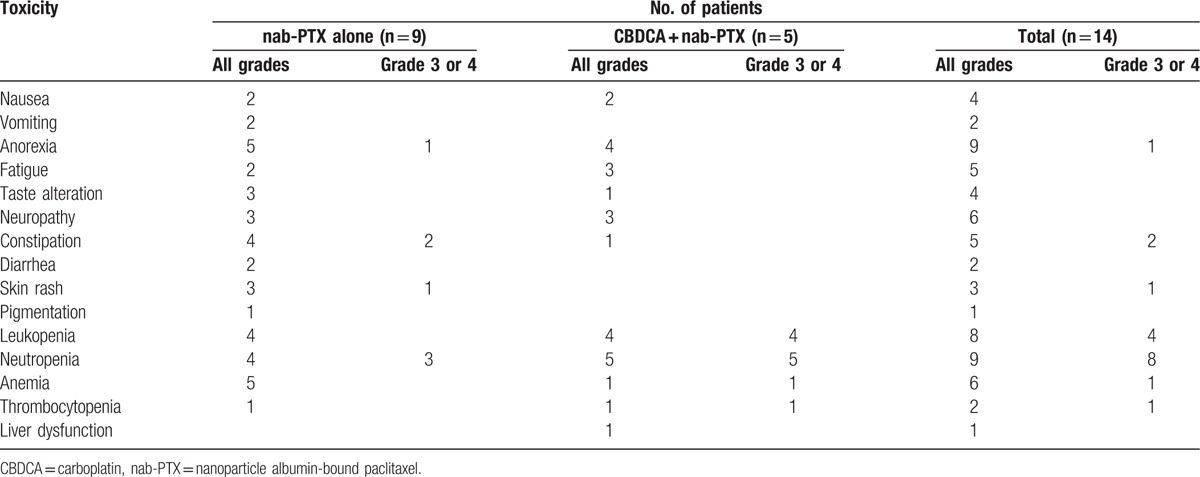
Toxicity of chemotherapy with nanoparticle albumin-bound paclitaxel alone and nanoparticle albumin-bound paclitaxel plus carboplatin in patients with relapsed small cell lung cancer included in the present study.

## 4 Discussion

This is the report about the efficacy and safety of nab-paclitaxel regimens for relapsed SCLC. Our results revealed RR, DCR, and median OS of 36%, 64%, and 7.8 months, respectively. Overall, chemotherapy with nab-paclitaxel regimens for previously treated, relapsed SCLC demonstrated moderate clinical efficacy and good tolerability.

Although there is no standard late-line regimen for the management of relapsed SCLC, some agents have been reported to be effective. In a phase III trial of topotecan for relapsed SCLC,^[2]^ RR was 7%, DCR was 51%, and the median OS was 6.5 months.^[2]^ In another study on amrubicin, RR, DCR, and the median OS were 33%, 78%, and 8.9 months, respectively.^[4]^ Japanese data have shown that amrubicin may be superior to topotecan, with acceptable toxicity, for relapsed SCLC.^[20]^ Another prospective analysis of late-line chemotherapy for SCLC^[7]^ showed that gemcitabine was associated with an RR of 13%, a DCR of 34%, and a median OS of 4 months. The values obtained for the nab-paclitaxel regimens used in the present study were comparable with these values. Considering the fact that 6 of the 14 patients exhibited ILD, whose prognosis were generally poor, our findings suggest that nab-paclitaxel regimens are suitable options for relapsed SCLC.

The outcomes of late-line chemotherapy for relapsed SCLC depend on several factors, such as the interval between cessation of first-line chemotherapy and relapse, the response to first-line chemotherapy, and previously administered regimens. Paclitaxel has shown an activity different from that of other drugs, either as first-line chemotherapy or salvage chemotherapy for SCLC.^[10,21,22]^ Therefore, paclitaxel and nab-paclitaxel regimens do not exhibit cross-resistance with etoposide, irinotecan, amrubicin, or topotecan, which are generally used to treat SCLC. From this point, they play an important role in SCLC treatment.

In the present study, weekly nab-paclitaxel administered as monotherapy for relapsed SCLC resulted in an RR of 11%, a DCR of 44%, and a median OS of 4 months. Weekly paclitaxel administration has shown similar results, with an RR of 24%, a DCR of 42%, and a median OS of 5.8 months, for a cohort of 21 patients with relapsed SCLC.^[8]^ Although weekly paclitaxel monotherapy demonstrated slightly better efficacy compared with nab-paclitaxel monotherapy, this difference is likely to be caused by variations in patient backgrounds. In the abovementioned phase II study,^[8]^ 16 of 21 patients received paclitaxel as second-line treatment, and half the patients exhibited sensitive relapse after first-line chemotherapy. On the contrary, all patients in our study received weekly nab-paclitaxel as third-line or later chemotherapy, and almost all exhibited refractory relapse. Tumors exhibiting sensitive relapse are associated with a higher probability of responding to later-line chemotherapy compared with those exhibiting refractory relapse, which may also be refractory to other drugs. In fact, the other report about weekly nab-paclitaxel monotherapy showed a response rate of 33% because more patients with sensitive relapse were included.^[16]^ In addition, the fact that 4 of 9 patients in the present study had previously received paclitaxel would have affected the clinical efficacy of nab-paclitaxel.

As mentioned above, nab-paclitaxel alone demonstrated moderate clinical efficacy in the present study. However, combination therapy with CBDCA showed high clinical efficacy, with a DCR of 100% and a median OS of 10.6 months. Although the patient backgrounds differed between the monotherapy and combination therapy groups, these findings are consistent with those of previous studies on paclitaxel for relapsed SCLC, where paclitaxel monotherapy resulted in an RR of 24% to 29% and a median OS of 3.3 to 5.8 months,^[8,10]^ and combination therapy with paclitaxel and CBDCA resulted in an RR of 25% to 79% and a median OS of 7.0 to 7.8 months.^[11,23]^ Although direct comparisons are difficult, we believe that paclitaxel regimens could be more effective when combined with CBDCA. Moreover, a preclinical study has suggested that paclitaxel interacts synergistically with CBDCA.^[24]^ Therefore, when paclitaxel or nab-paclitaxel regimens are selected for SCLC, combination with CBDCA is recommended when the general condition of patients is good, such as PS 0 to 1 at a young age.

The total rate of severe adverse events (grade 3 or 4) was low in the present study, and all adverse events were manageable. In particular, pneumonitis and acute exacerbation of ILD were not observed, despite the inclusion of 8 patients with comorbid pulmonary disease. These findings suggest that nab-paclitaxel regimens are well-tolerated. The frequency of severe neutropenia (grade 3 or 4) was higher in the combination therapy group, although it was resolved after treatment in all patients. This result is consistent with that of previous studies on nab-paclitaxel for SCLC.^[16,17]^ In a weekly paclitaxel monotherapy study, neuropathy was recorded for 12 of 21 patients (10 grade 1 or 2, two grade 3).^[8]^ In another study of paclitaxel and CBDCA, neuropathy occurred in 60% to 72% patients.^[11,23]^ In the present study, 6 of the 14 patients developed neuropathy, which was not severe in any patient. In previous studies as well, weekly nab-paclitaxel monotherapy and combination therapy with weekly nab-paclitaxel and CBDCA did not cause severe neuropathy.^[16,17]^ Similar to NSCLC patients, SCLC patients develop lesser toxicity with nab-paclitaxel regimens than with paclitaxel regimens, because nab-paclitaxel is administered weekly, whereas paclitaxel is administered tri-weekly (CBDCA plus paclitaxel).

## 5 Conclusions

The results of our study suggest a moderate clinical efficacy for nab-paclitaxel chemotherapy regimens for relapsed SCLC, with an acceptable safety and tolerability profile. In particular, combination with CBDCA showed high clinical efficacy. However, because the frequency of neutropenia was higher in the combination therapy, it may be necessary to consider adjustment of the administration schedule (dose reduction or skip of day15, etc) from the start of treatment. Although there is an ongoing clinical trial of monotherapy,^[25]^ other trials of schedule-adjusted combination therapy are also warranted to clarify the appropriate nab-paclitaxel regimen for relapsed SCLC.

## Acknowledgments

The authors wish to thank all the study participants and their families.
